# Potential Celiac Patients: A Model of Celiac Disease Pathogenesis

**DOI:** 10.1371/journal.pone.0021281

**Published:** 2011-07-08

**Authors:** Maria Pia Sperandeo, Antonella Tosco, Valentina Izzo, Francesca Tucci, Riccardo Troncone, Renata Auricchio, Jihane Romanos, Gosia Trynka, Salvatore Auricchio, Bana Jabri, Luigi Greco

**Affiliations:** 1 European Laboratory for Food Induced Disease, University of Naples Federico II, Naples, Italy; 2 Department of Genetics, University Medical Centre of Groningen, University of Groningen, Groningen, The Netherlands; 3 Department of Medicine, Department of Pathology and Department of Paediatrics, University of Chicago, Chicago, Illinois, United States of America; 4 Department of Pediatrics, University of Naples Federico II, Naples, Italy; Kyushu Institute of Technology, Japan

## Abstract

**Background and Aim:**

Potential celiacs have the ‘celiac type’ HLA, positive anti-transglutaminase antibodies but no damage at small intestinal mucosa. Only a minority of them develops mucosal lesion. More than 40 genes were associated to Celiac Disease (CD) but we still do not know how those pathways transform a genetically predisposed individual into an affected person. The aim of the study is to explore the genetic features of Potential CD individuals.

**Methods:**

127 ‘potential’ CD patients entered the study because of positive anti-tissue transglutaminase and no mucosal lesions; about 30% of those followed for four years become frankly celiac. They were genotyped for 13 polymorphisms of ‘candidate genes’ and compared to controls and celiacs. Moreover, 60 biopsy specimens were used for expression studies.

**Results:**

Potential CD bear a lighter HLA-related risk, compared to celiac (χ^2^ = 48.42; *p value* = 1×10^−8^). They share most of the polymorphisms of the celiacs, but the frequency of c-REL* G allele was suggestive for a difference compared to celiac (χ^2^ = 5.42; *p value* = 0.02). One marker of the KIAA1109/IL-2/IL-21 candidate region differentiated potentials from celiac (rs4374642: χ2 = 7.17, *p value* = 0.01). The expression of IL-21 was completely suppressed in potentials compared to celiacs (*p value* = 0.02) and to controls (*p value* = 0.02), in contrast IL-2, KIAA1109 and c-REL expression were over-expressed.

**Conclusions:**

Potential CD show genetic features slightly different from celiacs. Genetic and expression markers help to differentiate this condition. Potential CD is a precious biological model of the pathways leading to the small intestinal mucosal damage in genetically predisposed individuals.

## Introduction

The pathway of gluten-induced immunoresponse in Celiac Disease (CD) is not yet completely clarified to date, but a tremendous progress was gained recently [1], [2] by the discovery of candidate genes identified by genome wide association studies (GWAS) [3]–[5]. More than 40 candidate genes were discovered, each of them giving a minute contribution to the genomic variance in CD [6]. Only a functional profile of the gluten induced immunoresponse will shed light into the complex interaction of these molecules. The way to progress is to draw reliable pieces of functional pathways that may build up the puzzle of the gluten-induced immunoresponse. New genes, added to HLA, account for around 50% of the genetic risk. Even though relatively homogeneous, CD is probably not one disease, and different pathways and risk factors can be probably distinguished. The progressive stages of the disease are a resource to better understand the role of the genes identified up to date.

Alongside the HLA, a robust association was identified by GWAS on chromosome 4q27, containing the IL-2, IL-21 and KIAA1109 gene cluster [3]. We did confirm this strong association in our CD patients and controls, originated from the same region of Southern Italy (unpublished data). The discovery of a heritable variation in this gene cluster points to a direct involvement of these cytokines. In active CD there is an enhanced production of IL-21 [1]. The quiescence of the active phase of CD, obtained by a gluten free diet (GFD), reverts the IL-21 activity to normal values. When mucosal biopsies from treated CD patients are challenged with gluten peptides, IL-21 is again over expressed [1]. IL-21 induces, in active CD, T-bet expression [7]. The over expression of IL-21 is likely to play a crucial role in the activation of cytotoxic T-cell leading to epithelial cell death and mucosal destruction of active CD [8].

c-REL and TNFAIP3 are key mediators in the nuclear factor kappa B (NF-kB) inflammatory signalling pathway [5]. We suggested previously an early involvement of the NF-kB pathway in the gluten-induced inflammation either in acute CD as well as in gliadin exposed small intestinal mucosa tissue culture [9]. We proposed that, through the NF-kB pathway, gliadin peptides could stimulate iNOS expression in IFN-γ treated macrophages from non-celiac patients [10].

So far, genetic studies gave a solid base to explore biological models of gluten-induced immune response, but, no genotype-phenotype association could be found for any of the above listed candidate genes [11].

Recently the clinical presentation of CD underwent substantial changes: from classical malabsorption driven picture to light or absent symptoms associated to moderate damage of intestinal mucosa. The term ‘potential CD’ was assigned to individuals with proper DQ2 or DQ8, production of anti Transglutaminase (anti TTG) antibodies and normal small intestinal mucosa, classified as Marsh 0 (no damage) or Marsh 1 stage (unspecific intra epithelial infiltration only). These are also defined “serological positive cases” [12]–[14]. Potential patients suggest that the development of adaptive anti-gluten immunity is not sufficient to develop villous atrophy. This is supported by animal models showing that inflammatory anti-gluten responses are not sufficient to develop mucosal lesions [2]. Thus, we can subgroup individuals into three groups: controls, patients who developed antibodies but no intestinal lesions (M0 and M1 potentials) and patients with antibodies and villous atrophy (overt CD). Analyzing differences among these subgroups may shed light into the genes differentially involved in development of adaptive anti-gluten immunity (different between control and potential) and tissue lesions (different between potential and CD), as well as genes involved in all steps of CD pathogenesis (different between control and potential, and potential and CD).

We have a sizeable cohort of a living experimental model of CD: intestinal production of anti TTG antibodies but no specific mucosal damage. The vast majority of these individuals have no symptoms and may be grouped into low-medium-high anti-TTG producers, but all have a small intestinal mucosa with normal villi/crypt ratio, none or moderate IEL infiltration and normal epithelial layer. Over a prolonged follow up on gluten containing diet only about 1/3^rd^ develop mucosal damage, most frequently not associated to symptoms [14]. The reasons why potential CD do not show any degree of substantial mucosal damage, albeit the presence of all the features of CD, is the question, whose answer can contribute to the understanding of the pathogenesis of CD.

Our aim is to explore the presence of genetic and expression factors that may differentiate potential patients from overt CD patients.

## Results

### HLA typing

The HLA genotype of 127 potential CD cases was compared to that of 311 Celiacs previously reported by us [15]. Potential CD less frequently belong to high risk classes (double DQ2 or DQ2 in trans), while they show more frequently very low or moderate HLA-related risk (Table 1). We observed more cases in the HLA class at lower risk among potentials as compared to full celiacs: these cases bear half of the DQ2 heterodimer, either DQB1*02 or DQA1*05 only. To avoid a selection bias we did not excluded these potential cases, who produced anti TGASE and were anti Endomysium positive.

**Table 1:** HLA risk classes distribution.

HLA HaplotypeRisk Class	311 CD cases	127 PotentialCD cases
Double DQ2	74 (24%)	12 (9.4%)
DQ2 in trans	117 (38%)	23 (18.1%)
DQ2 single	79 (25%)	48 (37.8%)
DQ8 or DQB1*02 (DQA1*05 negative)	13 (4%)	21 (16.5%)
NoDQ2/NoDQ8	28 (9%)	23 (18.0%)
*χ^2^ = 48.42 p = 1×10^8^*		

When we compared the HLA risk between M0 and M1 potential cases we did not observe any difference (χ^2^ = 1.92; *p* = 0.75, data not shown). Cases who developed small intestinal atrophy during follow up did not show an HLA distribution different from those who remained potential (χ^2^ = 5.17; *p* = 0.27, data not shown).

### Eight Candidate Genes SNPs genotype and allele distribution

Hunt et al. [4] reported 8 new CD risk loci, located in different chromosomes. The size of the sample of the potential CD has no sufficient power to differentiate the polymorphisms distribution between potential CD and controls: nevertheless we found that three SNPs in LPP, c-REL and CCR regions respectively showed significant differences between controls and potential CD in their genotype distribution (Table 2). Seven out of 8 SNPs analyzed showed no significant differences between potential and CD cases in their genotype distribution. The SNP marking the c-REL gene showed the GG genotype 2.5 times more often in potentials versus ‘full’ CD and versus Controls (Table 2).

**Table 2:** Association results for 8 celiac non-HLA risk variants.

		Controls	Potential CD	χ^2^	*p value*	CD cases	Potential CD	χ^2^	*p value*
**RGS1 (rs2816316)**	AA	488 (68,6%)	88 (69.3%)	0.32	0.85	463 (72,7%)	88 (69.3%)	2.67	0.26
	AC	193 (27.1%)	35 (27.6%)			166 (26.1%)	35 (27.6%)		
	CC	30 (4.2%)	4 (3.1%)			8 (1.3%)	4 (3.1%)		
**IL18RAP (rs917997)**	AA	42 (5.9%)	7 (5.5%)	1.07	0.58	41 (6.4%)	7 (5.5%)	0.69	0.71
	AG	246 (34.6%)	50 (39.4%)			227 (35.6%)	50 (39.4%)		
	GG	423 (59.5%)	70 (55.1%)			369 (57.9%)	70 (55.1%)		
**LPP (rs1464510)**	AA	108 (15.2%)	28 (22.0%)	**7.58**	**0.02**	152 (23.9%)	28 (22.0%)	0.77	0.68
	AC	362 (50.9%)	70 (55.1%)			324 (50.9%)	70 (55.1%)		
	CC	241 (33.9%)	29 (22.8%)			161 (25.3%)	29 (22.8%)		
**OLIG3 (rs2327832)**	AA	480 (67.7%)	84 (66.7%)	0.05	0.98	399 (63.6%)	84 (66.7%)	0.81	0.67
	AG	202 (28.5%)	37 (29.4%)			208 (33.2%)	37 (29.4%)		
	GG	27 (3.8%)	5 (4.0%)			20 (3.2%)	5 (4.0%)		
**TAGAP (rs1738074)**	AA	125 (17.6%)	26 (20.5%)	0.6	0.74	144 (22.6%)	26 (20.5%)	0.36	0.84
	AG	354 (49.9%)	61 (48.0%)			305 (47.9%)	61 (48.0%)		
	GG	231 (32.5%)	40 (31.5%)			188 (29.5%)	40 (31.5%)		
**c-REL (rs842647)**	AA	404 (56.8%)	62 (48.8%)	**9.84**	**0.01**	359 (56.4%)	62 (48.8%)	**10.8**	**0.01**
	AG	272 (38.3%)	50 (39.4%)			249 (39.1%)	50 (39.4%)		
	GG	35 (4.9%)	15 (11.8%)			29 (4.6%)	15 (11.8%)		
**CCR (rs6441961)**	AA	99 (13.9%)	25 (19.7%)	**8.28**	**<0.01**	120 (18.8%)	25 (19.7%)	2.29	0.32
	AG	318 (44.7%)	66 (52.0%)			293 (46.0%)	66 (52.0%)		
	GG	294 (41.4%)	36 (28.3%)			224 (35.2%)	36 (28.3%)		
**SCHIP1 (rs17810546)**	AA	610 (85.8%)	102 (80.3%)	2.56	0.28	526 (82.6%)	102 (80.3%)	0.92	0.63
	AG	92 (12.9%)	23 (18.1%)			106 (16.6%)	23 (18.1%)		
	GG	9 (1.3%)	2 (1.6%)			5 (0.8%)	2 (1.6%)		

By analysis of allele distribution, we found that both LPP*A, c-REL*G, and CCR*A alleles were significantly associated to potential cases compared to controls (Table 3). Only c-REL*G allele was significantly associated with potential cases when compared to CD cases (Table 3).

**Table 3:** Association results of non-HLA SNPs alleles.

Potential CD and controls									
SNP	Locus	A1	A2	Pot CD Cases, Controls Ratios	MAF Pot CD cases	MAF controls	χ^2^	*p value*	Odds ratio (95% confidence interval)
rs2816316	RGS1	A	C	214∶44, 1169∶253	0.17	0.18	0.08	0.77	1.05 (0.74–1.50)
rs917997	IL18RAP	G	A	193∶65, 1092∶330	0.25	0.23	0.48	0.49	0.90 (0.66–1.22)
**rs1464510**	**LPP**	**C**	**A**	**129∶129, 844∶578**	**0.50**	**0.41**	**7.84**	**0.01**	**0.68 (0.52–0.89)**
rs2327832	OLIG3	A	G	207∶49, 1162∶256	0.19	0.18	0.17	0.68	0.93 (0.66–1.31)
rs1738074	TAGAP	G	A	143∶115, 816∶604	0.44	0.42	0.37	0.54	0.92 (0.70–1.20)
**rs842647**	**c-REL**	**A**	**G**	**178∶80, 1080∶342**	**0.31**	**0.24**	**5.62**	**0.02**	**0.70 (0.53–0.94)**
**rs6441961**	**CCR**	**G**	**A**	**139∶119, 906∶516**	**0.46**	**0.36**	**8.99**	**<0.01**	**0.66 (0.51–0.87)**
rs17810546	SCHIP1	A	G	231∶27, 1312∶110	0.10	0.08	2.17	0.14	0.72 (0.46–1.12)

Potential CD and CD patients									
SNP	Locus	A1	A2	Pot CD cases. CD cases Ratios	MAF Pot CD cases	MAF CD cases	χ^2^	*p value*	Odds ratio (95% confidence interval)
rs2816316	RGS1	A	C	214∶44, 1092∶182	0.17	0.14	1.31	0.25	0.81 (0.56–1.16)
rs917997	IL18RAP	G	A	193∶65, 965∶309	0.25	0.24	0.10	0.75	0.95 (0.70–1.29)
rs1464510	LPP	C	A	129∶129, 646∶628	0.50	0.49	0.04	0.83	0.97 (0.74–1.27)
rs2327832	OLIG3	A	G	207∶49, 1008∶248	0.19	0.20	0.05	0.82	1.04 (0.74–1.46)
rs1738074	TAGAP	G	A	143∶115, 681∶593	0.44	0.46	0.34	0.56	1.08 (0.83–1.42)
**rs842647**	**c-REL**	**A**	**G**	**178∶80, 967∶307**	**0.31**	**0.24**	**5.43**	**0.02**	**0.71 (0.53–0.95)**
rs6441961	CCR	G	A	139∶119, 741∶533	0.46	0.42	1.61	0.20	0.84 (0.64–1.10)
rs17810546	SCHIP1	A	G	231∶27, 1158∶116	0.10	0.09	0.47	0.49	0.84 (0.55–1.33)

None of the candidate genes distinguished between M0 and M1 potential cases (data not shown). M0 patients were not different from controls, except for CCR gene. M1 patients reinforced the significant differential distribution of the c-REL genotype when compared to CD: again the GG genotype was about three times more frequent in M1 patients compared to CD patients. The same trend, with good statistical significance, is observed between M1 potential and controls not only for c-REL but also for LPP gene (Table S1).

The eleven cases who developed small intestinal atrophy during follow up did not show an allelic and genotypic distribution different from those who remained potential, maybe because of the sample size that was too small to evaluate differences (Table S2 and S3).

### Five Candidate SNPs on the 4q genomic region

Despite the limited sample size we found that three SNPs in the 4q genomic region showed significant differences between controls and potential CD patients (rs4374642, rs13119723 and rs6840978) in their genotype distribution. In addition, we found also significant differences between potential CD and CD patients for three SNPs (rs4374642, rs13119723 and rs6822844) (Table 4).

**Table 4:** Association results for 5 celiac non-HLA risk variants, located on 4q27 chromosome.

		Controls	Potential CD	χ^2^	*p value*	CD cases	Potential CD	χ^2^	*p value*
**KIAA1109 (rs4374642)**	CC			**4.52**	**0.03**	4 (1.2%)		**7.22**	**0.03**
	CT	63 (14.2%)	9 (7.1%)			53 (15.3%)	9 (7.1%)		
	TT	381 (85.8%)	118 (92.9%)			290 (83.6%)	118 (92.9%)		
**KIAA1109 (rs13119723)**	AA	397 (88.6%)	104 (81.3%)	**5.99**	**0.05**	290 (84.1%)	104 (81.3%)	**8.16**	**0.02**
	AG	48 (10.7%)	21 (16.4%)			55 (15.9%)	21 (16.4%)		
	GG	3 (0.7%)	3 (2.3%)				3 (2.3%)		
**KIAA1109 (rs1127348)**	CC	16 (3.6%)	8 (6.3%)	2.05	0.36	9 (2.6%)	8 (6.3%)	3.51	0.17
	CT	122 (27.5%)	37 (29.1%)			103 (30.3%)	37 (29.1%)		
	TT	205 (68.8%)	82 (64.6%)			228 (67.1%)	82 (64.6%)		
**IL2/IL21 (rs6822844)**	GG	358 (80.1%)	103(79.8%)	0.35	0.84	267 (81.9%)	103(79.8%)	**7.61**	**0.02**
	GT	82 (18.3%)	23 (17.8%)			59 (18.1%)	23 (17.8%)		
	TT	7 (1.6%)	3 (2.3%)				3 (2.3%)		
**IL21 (rs6840978)**	CC	363 (81.6%)	85 (72.0%)	**17.94**	**<0.01**	222 (76.8%)	85 (72.0%)	4.5	0.10
	CT	82 (18.4%)	29 (24.6%)			65 (22.5%)	29 (24.6%)		
	TT		4 (3.4%)			2 (0.7%)	4 (3.4%)		

The rs4374642, rs13119723 and rs6840978 SNPs, respectively, were significantly associated when Potential CD cases were compared to Controls (Table 5). In contrast, only the rs4374642*T was the risk allele associated with potential cases when compared to CD cases (Table 5).

**Table 5:** Association results of non-HLA SNPs Alleles, located on chromosome 4q27.

Potential CD and Controls									
SNP	Locus	A1	A2	Pot CD Cases, Controls Ratios	MAF Pot CD cases	MAF Controls	χ^2^	*p value*	Odds ratio (95% confidence interval)
**rs4374642**	**KIAA1109**	**T**	**C**	**249∶9, 823∶63**	**0.03**	**0.07**	**4.44**	**0.03**	**2.12 (1.04–4.32)**
**rs13119723**	**KIAA1109**	**A**	**G**	**229∶27, 832∶54**	**0.10**	**0.06**	**5.97**	**0.01**	**0.55 (0.34–0.89)**
rs1127348	KIAA1109	T	C	201∶53, 732∶154	0.21	0.17	1.61	0.20	0.80 (0.56–1.13)
rs6822844	IL2/IL21	G	T	228∶28, 796∶96	0.11	0.10	0.01	0.94	0.98 (0.63–1.53)
**rs6840978**	**IL21**	**C**	**T**	**199∶37, 802∶82**	**0.16**	**0.09**	**8.04**	**<0.01**	**0.55 (0.36–0.83)**

Potential CD and CD patients									
SNP	Locus	A1	A2	Pot CD Cases, CD cases Ratios	MAF Pot CD cases	MAF CD cases	χ^2^	*p value*	Odds ratio (95% confidence interval)
**rs4374642**	**KIAA1109**	**T**	**C**	**249∶9, 622∶58**	**0.03**	**0.08**	**7.17**	**0.01**	**2.58 (1.26–5.29)**
rs13119723	KIAA1109	A	G	229∶27, 604∶54	0.10	0.08	1.25	0.26	0.76 (0.47–1.23)
rs1127348	KIAA1109	T	C	201∶53, 552∶120	0.21	0.18	1.10	0.29	0.82 (0.58–1.18)
rs6822844	IL2/IL21	G	T	228∶28, 592∶58	0.11	0.09	0.87	0.35	0.79 (0.49–1.28)
rs6840978	IL21	C	T	199∶37, 503∶69	0.16	0.12	1.92	0.17	0.74 (0.48–1.14)

Cases who developed small intestinal atrophy during follow up did not show an allelic and genotypic distribution different from those who remained potential (Table S2 and S3).

### Expression Studies

**Figure 1:**
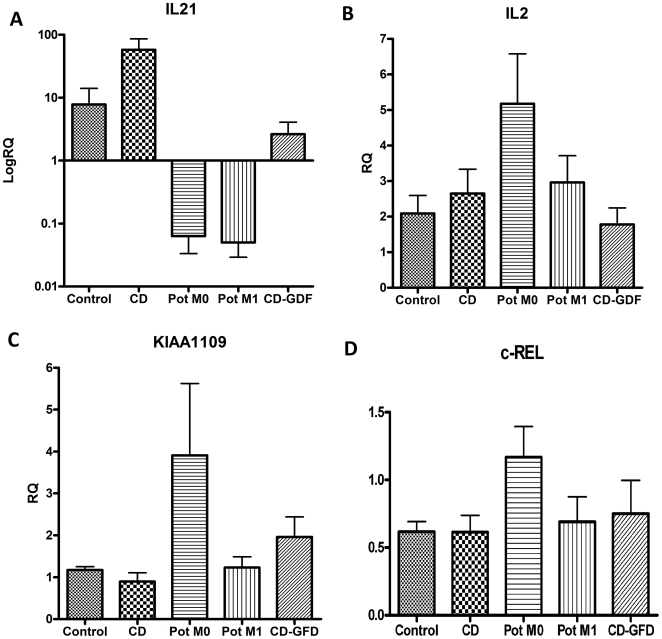
Expression studies of candidate genes on RNA extracted from small intestinal mucosal biopsies.

The expression of the IL-21 gene was completely suppressed in potential CD, as compared to CD (Mann-Whitney test; Rank test, *p*<0.01) and to controls (Fig. 1A). We underline that the values of expression in potential CD either completely normal as well as infiltrated were consistently lower that the controls (*p* = 0.02 for both stages). So IL-21 expression is markedly suppressed in these patients. Celiac patients revert completely IL-21 at the levels of controls after at least one year of GFD. But IL-21 expression in celiacs after GFD is still higher than that of the potential CD (*p* = 0.01).

IL-2 is over-expressed in M0 potential CD as compared to controls (*p* = 0.03) and to celiacs on GFD (*p* = 0.01) (Fig. 1B). Fig. 1C shows the expression of the KIAA1109 gene: its pattern is very similar to that of IL-2. It may be noted that M1 cases have an expression 4 times lower than M0 potentials (*p* = 0.04) but a marked increase over the controls (*p*<0.01) and of about 4 folds over the celiacs (*p* = 0.01). Celiacs on GFD show higher values than acute phase celiacs (*p* = 0.03) and controls (*p* = 0.02).

The expression of the c-REL gene was higher in M0 potentials, compared to CD cases (*p* = 0.04) and to controls (*p* = 0.03) (Fig. 1D).

We explored the relationship between the expression data with the genotype of the same patients for the same gene. We could not recover all DNA samples from the patients, whose RNA was used for expression experiments. Nevertheless we could not found a specific genotype related to the expression value for the same gene, as reported previously [5].

## Discussion

This work attempts to explore the genetic and expression profiles of a special set of individuals in the CD domain. Potential Celiac patients, with positive serology and undamaged small intestinal mucosa appear to be differently associated with some genetic risk factors compared to full blown CD patients.

Potential CD bears a load of HLA-related CD risk significantly lighter than that of CD cases, with a mix of high-intermediate-low risk different from that observed in CD cases. No difference in HLA profile was observed between M0 and M1 potential patients, neither between the cases who developed full blow disease and those who remained potential at follow up. Subjects without the classical DQ2 or DQ8 heterodimers were double in potential (23, 18.0%) than in classical celiacs (28, 9.0%), but they produced antibodies against Human Tissue Tranglutaminase, with positive Endomysial antibodies, just as much as the DQ2 positive cases. In addition we could find no discrimination on the outcome between the DQ2/DQ8 carriers and those who carried only half of the heterodimers. This is one of the results of this work and should not produce a selection bias. Three cases were questionable on the basis of having only DQA1*01 /DQB1*0501: but this haplotype was previously described in celiac disease [16]. Although they may not represent the typical potential celiac, we did not exclude them, to avoid selection bias. We better accept that only 3/127 cases may not show the full pattern of the majority of the cohort.

We identified six polymorphisms suggestive to be differently distributed between controls and potential CD: KIAA1109, IL-21, LPP, c-REL and CCR. We suppose that these factors are implicated at some stages of CD pathogenesis, and that, as for HLA, there may be a “gene-dosage effect”. Furthermore, c-REL and KIAA1109 are more expressed in M0 potential patients suggesting that they may play a role in the development of adaptive anti-gluten immunity.

The SNP at c-REL gene shows a different distribution of the G allele, as well as of the corresponding genotype, in potential CD versus celiacs and versus controls. Unfortunately we were unable to correlate the G allele of the polymorphic marker of the gene with RNA expression, but we will explore further in more cases. c-REL gene has been recently associated to CD but no significant effect of the rs842647 polymorphism on gene expression was observed [5]. In small-intestine tissue from controls and from treated and untreated celiac patients, c-REL gene did not show significant differential expression among the three groups [5]. c-REL, a subunit of the NF-kB complex, is crucial for the regulation of this major nuclear factor for the innate and adaptive immunity [17]. It's regulation can terminate the NF-kB activation by innate immune stimuli. The integral structure of c-REL is crucial for the activation as well as for the termination of NF-kB induced immunoresponse [18].

In conclusion, the finding of the different allele and genotype distribution of this gene in the potential CD model does point to the crucial role of NF-kB in the pathogenesis of the innate as well as induced immune response in CD. Sustained activation of NF-kB in intestinal mucosa of CD patients leads to prolonged induction of inflammatory genes expression and thereby perpetuates the chronic inflammatory process. Our group produced consistent evidence of the very early involvement of NF-kB mechanism in the gliadin-induced innate immune response in CD [9]. NF-kB is constitutively active in intestinal mucosa of patients with untreated CD and reverts to normal values when gluten is removed from the diet.

The polymorphic markers of the 4q candidate region show significant differences between potential CD and CD cases.

Potential CD show a marked suppression of IL-21, below the level of CD cases, celiacs on GFD and even of normal controls. IL-2 and KIAA1109, on the contrary were both over-expressed in M0 potential CD compared to M1, to celiacs and to controls. M1 cases show an IL-2 and KIAA1109 expression very similar to that of CD.

The finding that the IL-21 gene differentiates potential from controls suggests that it may be implicated also in the adaptive anti-gluten immune responses. This is compatible with findings indicating that IL-21 is produced by follicular helper cells and plays a critical role in B cell differentiation. However IL-21 is also implicated in tissue damage because of its ability to promote NK activity and block regulatory T cells.

In accordance with the role of IL-21 in tissue damage and its down regulation in the mucosa of potential CD patients we found that the IL-21/IL-2 candidate region differentiates potential from CD patients. Conversely, IL-2, which was found to be a critical growth factor for Foxp3+ regulatory T cells, is highly expressed at the transcriptional level in potential CD mucosa. Because Foxp3 regulatory T cells play a critical role in blocking effectors T cell functions, these finding suggests that the level of IL-2 may be a critical factor in the development of tissue damage.

Potential patients have most of the features of CD patients: they have similar genes. produce the same antibodies but they lack the final destructive phase of the gluten induced immunoresponse, the cytotoxic destruction of small intestinal mucosa. No clinical, serological or histological features could distinguish M0 from M1 patients up to now [14], for the first time we can speculate that potential CD are a mixture of two populations, with different non-HLA genetics and significant differential expression of some candidate genes.

Those potential cases who developed full blow mucosal damage on follow up, did not show any of the time 0 serologic markers worse than those who remained potential over a prolonged follow up. Similarly no genetic polymorphism, including HLA, could predict the outcome of these potential cases. We observed the same distribution of the HLA as well as of the non-HLA genes in the cases who eventually become full blown celiacs.

The limit of this study is the relatively short follow up time of this peculiar cohort of Potential CD cases: up to now there has been no possibility to identify any marker (clinical, environmental, serological or genetic) that could help to predict the time of conversion from predisposition to CD to full blown disease. In conclusion we cannot state that the cohort of potential celiacs who have again show an undamaged intestinal mucosa after 4 to 6 years of follow up, will not eventually develop it later in life. We can only observe that this group did not developed the full blown disease after 4–6 years. But the genetic and expression features point to the existence of a peculiar molecular profile, that may help to identify cases who do not have the complete molecular repertoire to develop the disease.

They may indeed be considered a live biological model of CD pathogenesis, where the process is, for some time or even forever, interrupted just before the final destructive phase.

## Materials and Methods

### Ethics statement

The Study Protocol was approved by the Ethics Committee of the University of Naples Federico II. Biopsies were taken during routine hospital admission requested for diagnostic purposes: parents or guardian gave their informed consent about the endoscopic procedures and the biopsy. Patients did not underwent blood or specimen sampling over and above those requested by the routine diagnostic procedures according to the ESPGHAN guidelines [19].

### Subjects

1. 127 children (76 females, median age 6 years and 6 months, range 18 months–16 years) were classified as ‘potential CD’ on the basis of:
  - Increased levels of anti-TTG (IgA anti Human Tissues Transglutaminase) and Anti-Endomisium positive. Serum EMA and anti TTG IgA were detected by indirect immunofluorescence and by enzyme-linked immunosorbent assay (ELISA) technique using a kit based on human recombinant antigen respectively (Eu-tTg IgA Kit, Eurospital, Trieste).
  - Architecturally normal small intestinal mucosa (M0 and M1) on at least four mucosa samples, to minimize the possible bias due to patchy lesions. Definition of the stage of lesion was obtained by Marsh stages modified by Oberhuber [14].

At the first biopsy, patients underwent clinical and laboratory assessment (CD-related auto antibodies, thyroid auto antibodies, nutritional parameters, bowel inflammatory indexes). 50/127 (39.4%) patients belong to at risk groups: 32 were first-degree relatives of celiac patients, 18 were affected by autoimmune diseases (11 type 1 diabetes and 7 thyroiditis). 105/127 (82.7%) were asymptomatic; 21/127 showed symptoms attributable to CD, 17 gastrointestinal (abdominal pain, weight loss, diarrhoea, failure to thrive) and 4 extra intestinal (2 short stature, one dilatative cardiomyopathy, one refractory anaemia). 21 symptomatic patients went on GFD, the others continued on a normal diet with a 6 monthly follow-up schedule. After two years they were re-biopsied. The second biopsy was anticipated if symptoms ensued.

The 21 patients on a GFD became negative for CD-related auto antibodies but 6 of them did not show any clinical response to GFD. 105/127 patients continued a gluten containing diet. During follow-up CD serology became negative in about 15% and antibody fluctuation was observed in 42% with transient negativity for anti-TTG values. Four patients developed autoimmune disorders (1 type 1 diabetes and 3 thyroiditis), 1 patient developed a vitamin K deficiency and began a GFD. 44/105 patients repeated a biopsy after 2 years of follow-up.13/44 (29.5%) developed mucosa damage, all the others were invariant at the control biopsy [14]. In conclusion, 35 children were considered celiac patients: 23 patients were put on a GFD at the beginning of the study because of symptoms, 1 patient was put on a GFD due to appearance of symptoms during the follow-up and 11 patients developed atrophy.

2. To evaluate genetic risk allele a cohort of 643 overt CD cases, and 711 healthy controls were compared to the 127 potential CD patients. Controls were randomly selected from a DNA bank representing the healthy population of the region, stratified for province. All originated from the same centre and the same geographical area. Genotyping of the 4q candidate gene was performed on a set of about 350 cases and about 450 controls from the above sample.
3. To evaluate the HLA-related risk the 127 potential cases were compared to a set of 311 overt cases from the same sample described above and reported in Bourgey et al. [15].

### Genotyping

HLA typing was performed using Eu-Gene Risk kit (EUROSPITAL). Cases were grouped into 5 HLA, as reported previously [15]. Thirteen SNPs were genotyped using TaqMan technology (Applied Biosystems) as described elsewhere [3], [20]. Reactions were performed on 7900HT Fast Real-Time PCR System (Applied Biosystems).

### Expression Studies

The expression of KIAA1109, IL-2 and IL-21 was analyzed in mucosal biopsy samples from 17 CD cases, 21 potential CD patients (7 M0 and 14 M1), 8 CD patients on GFD, and 14 controls using Real Time PCR by ‘Assays-On-Demand’ (Applied Biosystems). Controls underwent endoscopy for functional disorders, from these we excluded subjects with Helicobacter pylori infection, known to modify the expression of IL-21 [21]. The analysis of the expression of c-REL gene was performed in mucosal biopsy samples from 10 CD cases, 10 potential CD patients (5 M0 and 5 M1), and 10 controls.

### Statistical Analysis

Frequencies were compared by the χ^2^ test with.05 probability of the null hypothesis. Skewed variables were log transformed when required. Expression data were compared by a signed Rank test for independent samples. Data analysis was performed using: Prism (GraphPad Software, San Diego, CA), SPSS 15 (SPSS Inc., Chicago, IL, USA) and Haploview 4.1.

## Supporting Information

Table S1 **Comparison between cases with completely normal mucosa (M0) and those with infiltrated mucosa (M1).**
(DOCX)

Table S2 **Association results of 13 non-HLA SNPs alleles.**
(DOC)

Table S3 **Association results for 13 celiac non-HLA risk variants.**
(DOC)
