# Supplementary material for: Potential Celiac Patients: A Model of Celiac Disease Pathogenesis
Source: PLoS One. 2011 Jul 8;6(7):e21281. doi: 10.1371/journal.pone.0021281 (PMC3132737; doi:10.1371/journal.pone.0021281)
Supplement: Table S1 — Comparison between cases with completely normal mucosa (M0) and those with infiltrated mucosa (M1). (DOCX) [file pone.0021281.s001.docx]

| **Supplementary Table S1** | | | | | | | | | | | |
| --- | --- | --- | --- | --- | --- | --- | --- | --- | --- | --- | --- |
|  |  | **Controls** | **Potential M0** | **Potential M1** | **CD cases** | **M0/Controls** | | **M1/CD** | | **M1/Controls** | |
|  |  |  |  |  |  | **χ^2^** | ***p*** | **χ^2^** | ***p*** | **χ^2^** | ***p*** |
| **c-REL**  **(rs842647)** | **AA** | 404 (56.8%) | 26 (47.3%) | 33 (47.8%) | 359 (56.4%) | 2.91 | 0.23 | **11.98** | **<0.01** | **10.9** | **<0.01** |
|  | **AG** | 272 (38.3%) | 24 (43.6%) | 26 (37.7%) | 249 (39.1%) |  |  |  |  |  |  |
|  | **GG** | 35 (4.9%) | 5 (9.1%) | 10 (14.5%) | 29 (4.6%) |  |  |  |  |  |  |
|  |  |  |  |  |  |  |  |  |  |  |  |
| **CCR**  **(rs6441961)** | **AA** | 99 (13.9%) | 11 (23.4%) | 9 (15.8%) | 120 (18.8%) | **6.12** | **0.04** | 1.20 | 0.55 | 4.22 | 0.12 |
|  | **AG** | 318 (44.7%) | 24 (51.1%) | 30 (52.6%) | 293 (46.0%) |  |  |  |  |  |  |
|  | **GG** | 294 (41.4%) | 12 (25.5%) | 18 (31.6%) | 224 (35.2%) |  |  |  |  |  |  |
|  |  |  |  |  |  |  |  |  |  |  |  |
| **LPP**  **(rs1464510)** | **AA** | 108 (15.2%) | 12 (21.8%) | 15 (21.7%) | 152 (23.9%) | 2.10 | 0.35 | 1.35 | 0.51 | **5.88** | **0.05** |
|  | **AC** | 362 (50.9%) | 28 (50.9%) | 40 (58.0%) | 324 (50.9%) |  |  |  |  |  |  |
|  | **CC** | 241 (33.9%) | 15 (27.3%) | 14 (20.3%) | 161 (25.3%) |  |  |  |  |  |  |
