# Supplementary material for: Potential Celiac Patients: A Model of Celiac Disease Pathogenesis
Source: PLoS One. 2011 Jul 8;6(7):e21281. doi: 10.1371/journal.pone.0021281 (PMC3132737; doi:10.1371/journal.pone.0021281)
Supplement: Table S2 — Association results of 13 non-HLA SNPs alleles. (DOC) [file pone.0021281.s002.doc]

| **Supplementary Table S2** | | | | | | | | | |
| --- | --- | --- | --- | --- | --- | --- | --- | --- | --- |
| Potential CD which develop villous atrophy and still Potential CD cases | | | | | | | | | |
| **SNP** | **Locus** | **A1** | **A2** | **Pot CD with atrophy,**  **Still Pot Ratios** | **MAF**  **Pot CD with atrophy** | **MAF**  **Still Potential CD** | **χ^2^** | **p value** | **Odds ratio**  **(95% confidence interval)** |
| rs2816316 | RGS1 | A | C | 16:6, 195:37 | 0.27 | 0.15 | 1.83 | 0.17 | 0.51 (0.18-1.38) |
| rs917997 | IL18RAP | G | A | 18:4, 172:60 | 0.18 | 0.25 | 0.63 | 0.43 | 1.57 (0.51-4.82) |
| rs1464510 | LPP | A | C | 12:10, 114:118 | 0.45 | 0.49 | 0.23 | 0.63 | 1.24 (0.52-2.99) |
| **rs2327832** | **OLIG3** | **A** | **G** | **14:8, 191:39** | **0.36** | **0.16** | **4.98** | **0.02** | **0.36 (0.14-0.91)** |
| rs1738074 | TAGAP | G | A | 13:9, 128:104 | 0.40 | 0.44 | 0.12 | 0.72 | 1.17 (0.48-2.85) |
| rs842647 | c-REL | A | G | 15:7, 159:73 | 0.31 | 0.31 | 0.01 | 0.97 | 0.98 (0.38-2.52) |
| rs6441961 | CCR | G | A | 14:8, 124:108 | 0.36 | 0.46 | 0.84 | 0.36 | 1.52 (0.61-3.78) |
| rs17810546 | SCHIP1 | A | G | 20:2, 207:25 | 0.09 | 0.10 | 0.06 | 0.80 | 1.21 (0.27-5.48) |
| rs4374642 | KIAA1109 | T | C | 22:0, 219:9 | 0.00 | 0.03 | 0.90 | 0.34 | 1.95 (1.10-34.61) |
| rs13119723 | KIAA1109 | A | G | 21:1, 204:26 | 0.04 | 0.11 | 0.96 | 0.33 | 2.67 (0.34-20.74) |
| rs1127348 | KIAA1109 | T | C | 18:4, 179:49 | 0.18 | 0.21 | 0.13 | 0.72 | 1.23 (0.40-3.81) |
| rs6822844 | IL2/IL21 | G | T | 21:1, 204:26 | 0.04 | 0.11 | 0.96 | 0.33 | 2.68 (0.34-20.74) |
| rs6840978 | IL21 | C | T | 14:2, 182:34 | 0.12 | 0.14 | 0.12 | 0.73 | 1.31 (0.28-6.02) |
