# Supplementary material for: Potential Celiac Patients: A Model of Celiac Disease Pathogenesis
Source: PLoS One. 2011 Jul 8;6(7):e21281. doi: 10.1371/journal.pone.0021281 (PMC3132737; doi:10.1371/journal.pone.0021281)
Supplement: Table S3 — Association results for 13 celiac non-HLA risk variants. (DOC) [file pone.0021281.s003.doc]

| **Supplementary Table S3** | | | | | |
| --- | --- | --- | --- | --- | --- |
|  |  | **116 Still Potential cases** | **11 Potential cases with CD phenotype** | **χ^2^** | ***p value*** |
| **RGS1**  **(rs2816316)** | AA | 82 (70.7%) | 6 (54.5%) | 2.07 | 0.36 |
|  | AC | 31 (26.7%) | 4 (36.4%) |  |  |
|  | CC | 3 (2.6%) | 1 (9.1%) |  |  |
|  |  |  |  |  |  |
| **IL18RAP**  **(rs917997)** | AA | 7 (6.0%) |  | 0.85 | 0.65 |
|  | AG | 46 (39.7%) | 4 (36.4%) |  |  |
|  | GG | 63 (54.3%) | 7 (63.6%) |  |  |
|  |  |  |  |  |  |
| **LPP**  **(rs1464510)** | AA | 24 (20.7%) | 4 (36.4%) | 1.99 | 0.37 |
|  | AC | 66 (56.9%) | 4 (36.4%) |  |  |
|  | CC | 26 (22.4%) | 3 (27.3%) |  |  |
|  |  |  |  |  |  |
| **OLIG3**  **(rs2327832)** | AA | 80 (69.6%) | 4 (36.4%) | 5.06 | 0.08 |
|  | AG | 31 (27.0%) | 6 (54.5%) |  |  |
|  | GG | 4 (3.5%) | 1 (9.1%) |  |  |
|  |  |  |  |  |  |
| **TAGAP**  **(rs1738074)** | AA | 25 (21.6%) | 1 (9.1%) | 1.44 | 0.49 |
|  | AG | 54 (46.6%) | 7 (63.6%) |  |  |
|  | GG | 37 (31.9%) | 3 (27.3%) |  |  |
|  |  |  |  |  |  |
| **c-REL**  **(rs842647)** | AA | 58 (50.0%) | 4 (36.4%) | 3.61 | 0.16 |
|  | AG | 43 (37.1%) | 7 (63.6%) |  |  |
|  | GG | 15 (12.9%) |  |  |  |
|  |  |  |  |  |  |
| **CCR**  **(rs6441961)** | AA | 23 (19.8%) | 2 (18.2%) | 1.82 | 0.40 |
|  | AG | 62 (53.4%) | 4 (36.4%) |  |  |
|  | GG | 31 (26.7%) | 5 (45.5%) |  |  |
|  |  |  |  |  |  |
| **SCHIP1**  **(rs17810546)** | AA | 93 (80.2%) | 9 (81.8%) | 0.19 | 0.91 |
|  | AG | 21 (18.1%) | 2 (18.2%) |  |  |
|  | GG | 2 (1.7%) |  |  |  |
|  |  |  |  |  |  |
| **KIAA1109**  **(rs4374642)** | CC |  |  | 0.94 | 0.40 |
|  | CT | 9 (7.9%) |  |  |  |
|  | TT | 105 (92.1%) | 11 (100%) |  |  |
|  |  |  |  |  |  |
| **KIAA1109**  **(rs13119723)** | AA | 92 (80.0%) | 10 (90.9%) | 0.85 | 0.65 |
|  | AG | 20 (17.4%) | 1 (9.1%) |  |  |
|  | GG | 3 (2.6%) |  |  |  |
|  |  |  |  |  |  |
| **KIAA1109**  **(rs1127348)** | CC | 8 (7.0%) |  | 0.96 | 0.62 |
|  | CT | 33 (28.9%) | 4 (36.4%) |  |  |
|  | TT | 73 (64.0%) | 7 (63.6%) |  |  |
|  |  |  |  |  |  |
| **IL2/IL21**  **(rs6822844)** | GG | 92 (79.3%) | 10 (90.9%) | 0.92 | 0.63 |
|  | GT | 21 (18.1%) | 1 (9.1%) |  |  |
|  | TT | 3 (2.6%) |  |  |  |
|  |  |  |  |  |  |
| **IL21**  **(rs6840978)** | CC | 78 (72.2%) | 6 (75.0%) | 0.31 | 0.86 |
|  | CT | 26 (24.1%) | 2 (25.0%) |  |  |
|  | TT | 4 (3.7%) |  |  |  |
|  |  |  |  |  |  |
